# Advancing HBV Diagnostics: The Role of Ultrasensitive HBsAg Testing

**DOI:** 10.3390/diagnostics15212744

**Published:** 2025-10-29

**Authors:** Hussain Ali, Carsten Buenning, David Daghfal

**Affiliations:** Abbott Diagnostics, Abbott Park, IL 60064, USA; carsten.buenning@abbott.com (C.B.); david.daghfal@abbott.com (D.D.)

**Keywords:** hepatitis B virus, chronic hepatitis infection, HBV diagnostics, diagnostic sensitivity, lower limit of detection, functional cure, early detection, therapeutic monitoring, HBV elimination

## Abstract

Hepatitis B virus (HBV) represents a significant global health challenge, affecting over 254 million individuals and contributing to 1.1 million deaths from liver-related complications in 2022. The World Health Organization has set ambitious targets to reduce HBV infections and mortality by 2030. However, only a small proportion (13%) of infected individuals receives timely diagnosis and treatment. HBV elimination efforts necessitate substantial improvements in HBV diagnosis, particularly in identifying early-stage infections, occult HBV infections (OBI), and breakthrough cases. The hepatitis B surface antigen (HBsAg) is a key biomarker in HBV diagnosis, serving as a reliable indicator of infection status and treatment response. Conventional HBsAg assays, with a lower limit of detection (LoD) between 0.03 and 250 IU/mL, often fail to detect OBI and HBV reactivation. In contrast, ultrasensitive HBsAg assays, with an LoD as low as 0.005 IU/mL, can improve the identification of low concentration levels of HBsAg, facilitating earlier diagnosis, monitoring of therapeutic response, and assessment for functional cure. Research confirms the superiority of ultrasensitive assays in detecting HBV in cases missed by conventional assays, detecting NAT-yield samples, and enabling earlier detection of HBV reactivation. This review examines the challenges in HBV diagnostics and the clinical utility of ultrasensitive HBsAg assays in improving progress toward global HBV elimination.

## 1. Introduction

Hepatitis B virus (HBV) remains a critical global health challenge, with over 254 million individuals living with chronic infection in 2022 and 1.2 million new infections reported annually [1]. The virus contributes to substantial morbidity and mortality from liver-related complications, such as cirrhosis and hepatocellular carcinoma (HCC), causing 1.1 million deaths in 2022 [1]. Alarmingly, only 13% of individuals with hepatitis B are aware of their infection, and just 3% of those with chronic hepatitis B (CHB) receive treatment [1]. Recognizing this urgency, the World Health Organization (WHO) set targets to reduce new infections by 90% and deaths by 65% between 2016 and 2030 [2]. However, progress has been hindered by lack of accurate and sensitive diagnostic assays, limited uptake of testing, and the COVID-19 pandemic, which put a strain on national healthcare systems [3,4]. If the current trajectory continues, annual global deaths from HBV could increase by 39% by 2030 [5], underscoring the need for a renewed focus on early detection and management.

Diagnostics are integral to HBV elimination efforts: early and accurate detection helps to decrease spread and initiate suppressive treatment [6]. Conventional biomarkers used in HBV diagnosis include HBV DNA, HBV surface antigen (HBsAg), HBV e antigen (HBeAg), antibody to the HBV core antigen (anti-HBc), and antibody to the HBV surface antigen (anti-HBs) [7]. While HBV DNA is the gold-standard marker of viral replication and occult HBV infection (OBI), HBsAg offers complementary and practical advantages [8,9]. HBsAg has long been a reliable serological marker for predicting clinical and therapeutic outcomes, with increased assay sensitivity enabling earlier detection and reducing the diagnostic window [10]. Notably, HBsAg testing is the primary recommended method for diagnosing CHB, and guidelines endorse it to characterize disease phase and guide management [8,11,12]. Its significance has further increased with the growing focus on functional cure (sustained HBsAg loss) in clinical practice [13].

Despite these advantages, limitations of conventional HBsAg assays remain. Current commercial serological assays with a sensitivity of 0.05 IU/mL may still miss infections in the early window period or cases of apparent HBsAg loss [10]. Challenges persist in detecting mutants, OBIs, HBV reactivations (HBV-R), and breakthrough infections. Emerging ultrasensitive HBsAg assays can address several of these limitations by detecting low antigen levels and identifying OBIs and residual viremia in patients previously considered cured [3,10,14]. Beyond enhancing screening, these assays can help identify true infection status, facilitating assessment of HBV-R risk and allowing physicians to confidently determine achievement of functional cure [10,14]. Despite their clinical importance, comprehensive reviews on ultrasensitive HBsAg assays are scarce.

Therefore, this review examines the current challenges in HBV diagnosis and the need for enhanced assay sensitivity. It focuses on ultrasensitive HBsAg assays and their role in addressing gaps in detection, monitoring, and therapeutic decision-making.

## 2. Challenges in HBV Diagnosis

### 2.1. Global HBV Burden

The global burden of HBV exhibits significant geographic variation, with transmission routes influenced by regional prevalence [6,15]. High-endemic regions, such as parts of Asia, Sub-Saharan Africa, and the South Pacific, experience mostly perinatal or early childhood transmission. Intermediate-endemic areas, including the Mediterranean, Alaska, and India, see similar early-life transmission, with additional spread through sexual contact, intravenous drug use, and unsafe injections in adulthood. In low-endemic regions, primarily in the developed world, transmission occurs mainly through sexual contact and intravenous drug use and among homeless and incarcerated individuals [6,15]. HBV is further categorized into distinct genotypes (A–H), each with specific geographic distributions. Multiethnic populations may harbor multiple genotypes [15].

Approximately 90% of infants born to HBsAg-positive or HBeAg-positive mothers are at risk of developing CHB, presenting a significant global public health concern [6]. Moreover, HBV accounts for 50–80% of HCC cases, making it a major contributor to global morbidity and mortality. HCC is the sixth-most common cancer and fourth-leading cause of cancer-related deaths worldwide [6]. To address this, the WHO set ambitious targets to eliminate HBV as a public health threat by 2030 [2]. However, the COVID-19 pandemic severely disrupted the cascade of viral hepatitis care, reducing consultation and referral of new patients by 31%. Additionally, HBsAg carriers received less stringent follow-up, with HBsAg and HBV DNA testing decreasing by 31% and 39%, respectively [4]. The pandemic’s economic effects also put additional pressure on public health initiatives [4]. A 1-year delay in viral hepatitis elimination programs is anticipated to increase HCC cases by 44,800 and liver-related deaths by 79,400 between 2020 and 2030 (relative to no delay) [16]. Other challenges include complexities in patient selection, such as identifying individuals who require antiviral therapy, and the need for optimal drug regimens.

Disparities in access to vaccination, diagnostics, and treatments and limited awareness, particularly in resource-limited settings, further exacerbate the situation [17]. Expanding access to high-sensitivity HBV diagnostics, particularly in resource-limited settings, is essential to improve early detection.

### 2.2. Limitations of Current Diagnostic Tools

Conventional biomarkers of HBV infection include HBV DNA, HBsAg, HBeAg, anti-HBc, and anti-HBs. These play a critical role in screening and therapeutic decision-making, including the initiation and monitoring of antiviral therapy [7]. Table 1 summarizes the interpretation of key serological markers across different stages of HBV infection, as indicated by the Centers for Disease Control and Prevention (CDC) and WHO [18,19].

Traditional assays often lack the sensitivity to identify certain phases of HBV infection, limiting their efficacy in guiding timely therapeutic interventions [20]. Early and accurate detection is particularly challenging in high-risk populations, such as immunocompromised individuals or those with OBI or HBV-R [20,21,22,23]. OBI refers to the persistence of viral genomes in hepatic nuclei, with an absence of detectable HBsAg and presence of very low levels of serum HBV DNA [3,24]. In such cases, common serological tests may be unreliable owing to low or intermittent HBV replication, and liver histology may be impractical due to its invasive nature [25]. The negative HBsAg findings may also be attributable to low circulating concentrations of HBsAg, low sensitivity of assays, HBsAg gene mutations, including immune-escape mutations in the S gene, or masking of HBsAg by anti-HBs [3,20,24,26,27].

HBV-R is characterized by the reappearance of HBV DNA and/or HBsAg seroreversion in individuals with resolved HBV or rise in viral load in patients with CHB [28]. HBV-R can occur spontaneously or be induced by immunosuppressive medication, chemotherapy, or biological agents that target immunological pathways. The current reported rate of HBV-R varies across studies, ranging from 10% to 16.9% or more [29,30]. A retrospective evaluation of patients with hematopoietic cell transplantation revealed a cumulative incidence of 9%, 21.7%, and 42.9% at 1, 2, and 4 years, respectively [31]. HBV-R can result in asymptomatic viral replication or even acute liver failure and death [32].

At present, early detection is the most effective measure to mitigate complications of HBV-R [29]. As intrahepatic HBV DNA can produce small amounts of HBsAg, monitoring HBsAg could help detect CHB early or reclassify a “cured” status to a “low-HBsAg-level chronic carrier” status [3,14]. Therefore, highly sensitive and highly specific diagnostic tools with a lower limit of detection (LoD) are essential not only to identify active infections but also to monitor treatment responses and determine therapy endpoints [3,10].

## 3. Paradigm Shift in HBV Treatment Goals

### 3.1. Treatment Endpoints

Conventional treatment endpoints are based on assessment of viral suppression, biochemical response, serologic response, and histologic improvement [13]. The management of CHB has traditionally focused on eliminating or suppressing HBV replication to prevent disease progression to cirrhosis and HCC [33]. While HBV DNA levels have served as the gold standard for treatment monitoring, undetectable levels do not reliably predict sustained remission. The optimal level of HBV DNA suppression that should be attained to achieve these benefits is not well defined, and more than 50% of patients relapse upon discontinuation of nucleos(t)ide analog (NA) therapy [13,34]. Therefore, undetectable HBV DNA by itself may be inadequate to predict treatment durability compared to biomarkers like HBsAg [13].

Biochemical response, indicated by aminotransferase normalization, is another classic endpoint in HBV treatment. Although associated with a reduced risk of complications, its utility as a therapy endpoint is limited by its non-specificity for CHB, inability to reflect disease severity, and variability in the upper limit of normal between different laboratory methods and trials [13]. In HBeAg-positive patients, spontaneous reduction or loss of HBeAg is a positive indication of immune suppression of the virus, but liver disease progression can still occur [13]. Histologic improvements, including fibrosis regression, can be achieved with prolonged antiviral therapy. However, biopsies have been replaced with noninvasive tests in clinical practice (serum fibrosis biomarkers, transient elastography), which primarily evaluate fibrosis severity rather than necro-inflammatory activity and require further research [13].

Given these limitations, a more precise and clinically relevant treatment endpoint is needed to guide therapeutic strategies and improve long-term outcomes.

### 3.2. Functional Cure and Emerging Focus on HBsAg

A 2016 workshop by the American Association for the Study of Liver Diseases (AASLD) and European Association for the Study of the Liver (EASL) on HBV treatment endpoints classified potential HBV cure as sterilizing, functional, and partial (Table 2) [35,36,37].

Although the ultimate therapeutic goal is a sterilizing cure, complete eradication of HBV covalently closed circular DNA (cccDNA) and integrated HBV DNA from infected cells is currently impossible [38]. Sterilizing cure has not yet been observed naturally in individuals with CHB or those who have recovered from acute infection. Further, no available treatment can reliably eliminate cccDNA [35]. While research into cccDNA-silencing compounds is ongoing, targeting a functional cure remains the most realistic and clinically relevant option [35,38].

The current most reliable indicator of functional cure is sustained HBsAg loss, confirmed at least twice over 6 months, with undetectable HBV DNA [13]. It captures both virological (undetectable serum HBV DNA) and clinical aspects (improved clinical outcomes) of functional cure [37]. Assessing HBsAg loss allows clinicians to safely discontinue antiviral therapy [34]. However, it is rarely achieved with current treatments. In patients receiving NA therapy, functional cure is observed in fewer than 10% of cases after a decade of continuous treatment. Similarly, pegylated interferon therapy has demonstrated HBsAg loss rates of 2–3% at the end of treatment (EoT), increasing to 8–14% over 3–5 years of post-treatment follow-up [39,40,41]. The risk of HCC persists even after HBsAg loss in patients older than 50 years or those with cirrhosis or coinfection with hepatitis C or D virus [42].

Despite these limitations, functional cure remains a feasible endpoint, particularly when achieved at a younger age and in the absence of significant fibrosis, and lowers risk of HCC [34]. Functional cure is being increasingly recognized as a key endpoint in determining long-term prognosis, guiding therapy cessation, and monitoring disease progression. Importantly, HBsAg seroclearance can remove the stigma linked to HBV infection, easing social and professional restrictions and representing a major milestone from a patient’s perspective [43].

The 2019 EASL-AASLD HBV Treatment Endpoints Conference suggested using functional cure as a primary endpoint of phase 3 trials, with HBsAg loss in ≥ 30% patients after 1 year of therapy voted as the desired response rate by over two-thirds of the participants [37]. However, the lower LoD of several available immunoassays is 0.05 IU/mL. With the paradigm shift in HBV treatment goals, there is a growing emphasis on lowering the HBsAg threshold to detect even minute quantities of the viral antigen, enabling better understanding of the HBsAg status prior to treatment cessation [44].

## 4. HBsAg: A Key Biomarker in HBV Diagnosis

HBsAg, a component of the viral envelope, is widely regarded as the “hallmark of HBV infection.” It is the first serological marker to appear in the blood after infection [14,45]. Its levels reflect the transcriptional activity of cccDNA in the liver [14,45] and vary significantly in the different phases of HBV infection, helping to track the natural history of the infection [46,47]. Further, the quantity of HBsAg in circulation is approximately 1000- to 10,000-fold higher than that of complete DNA-containing virus particles, making HBsAg a simple, reliable, and highly sensitive marker [46,48,49].

### 4.1. Current Landscape of HBsAg Assessment

The importance of HBsAg testing is reaffirmed in the most recent clinical practice guidelines on hepatitis management by the EASL (2025), WHO (2024), and CDC (2023) [8,12,50]. These guidelines strongly recommend HBsAg testing for the initial screening of HBV infection, including in high-priority groups, such as organ donors, healthcare workers, and pregnant women [8,50]. A positive HBsAg result guides subsequent serological and virological diagnostics, liver disease assessment, imaging studies, and biopsy [8,50]. These guidelines highlight the role of HBsAg testing throughout the disease continuum, including patient stratification, disease monitoring, treatment planning (initiation, use of combination therapy, monitoring, and cessation), long-term surveillance, and HBV-R risk assessment [8,50]. They also indicate the role of HBsAg testing in determining the need for vaccination and managing special populations, such as HIV-positive individuals, transplant recipients, and newborns [8,50].

Patients with acute infection typically exhibit HBsAg levels > 4 log10 IU/mL, while lower levels (<4 log10 IU/mL) are linked to CHB [7]. Similarly, persistent HBsAg detection beyond 6 months indicates CHB, whereas its absence suggests recovery from acute infection [49]. Evaluation of HBsAg kinetics also holds prognostic value and can help predict likelihood of HBsAg loss and risk of liver fibrosis and HCC [7,45]. Low baseline HBsAg (<50 IU/mL) and a significant decline during therapy (<70 IU/mL by week 8 of treatment) predict a good treatment response with HBsAg clearance [7]. Clearance of HBsAg, whether spontaneous or treatment-induced, represents a “functional cure” of HBV infection; however, the risk of HCC persists [7].

### 4.2. Ultrasensitive HBsAg Assays

A recent advance in HBV diagnosis, ultrasensitive HBsAg assays, have a lower LoD (0.005 IU/mL), allowing early detection of acute infection, HBsAg clearance, HBV-R or subclinical levels of HBsAg following seroclearance, and OBI [20,49].

Commercial ultrasensitive HBsAg assays include HBsAg NEXT Qualitative assay (Abbott Diagnostics, IL, USA), HBsAg NEXT Confirmatory assay (Abbott Diagnostics, IL, USA), and iTACT-HBsAg (Fujirebio Inc., Tokyo, Japan), among others [20,51].

#### Assay Platforms

HBsAg detection spans several assays formats, including chemiluminescent immunoassays (CLIA), lateral flow assays, and enhanced enzyme-linked immunoassays. A comparative analysis of these platforms is presented in Table 3 [3,10,52,53,54,55,56,57,58].

CLIAs represent the state-of-the-art in HBsAg detection. In these assays, the sample is combined with assay-specific diluents, anti-HBs coated paramagnetic microparticles, and an acridinium-labeled anti-HBs conjugate. This reaction mixture is then incubated. If HBsAg is present, the sample binds to the anti-HBs coated microparticles and labeled anti-HBs conjugate. Following a series of wash cycles, pre-trigger and trigger solutions are added, resulting in a chemiluminescent reaction [3,59]. These assays incorporate a specific diluent to enhance signal intensity and sensitivity and a goat polyclonal antibody to improve signal stability. Further, an additional wash step is included to improve specificity [7,59]. The automation, high throughput, high reproducibility, and less turnaround time make CLIAs highly suitable for screening of blood donors and large cohorts as well as for monitoring of treatment efficacy [3,10,57,58,59].

## 5. Clinical Role of Ultrasensitive HBsAg Assays

### 5.1. Improved Sensitivity and Specificity

Sickinger et al. compared the sensitivity of an ultrasensitive immunoassay with that of an on-market HBsAg qualitative/confirmatory assay [48] using 450 HBsAg PCR-positive specimens from patients with acute and chronic infections. The analytical sensitivity of the ultrasensitive and comparator assays ranged between 0.004 and 0.006 IU/mL and 0.017 and 0.022 IU/mL, respectively, and their clinical sensitivity was 100.00% and 99.78%, respectively. The four-fold increased sensitivity enhances clinical diagnosis and screening, mutant detection, and seroconversion sensitivity [48]. Consistent with these findings, another study showed that the HBsAg detection rate increased by 6–7% when compared with conventional assays [60].

Lou et al. compared the sensitivity of an ultrasensitive assay with that of other commercially available assays and found 3.2- to 7.1-fold greater sensitivity. Of 27 seroconversion panels included in the study, the investigational assay detected more panel members (191 of 364) than the comparator assays (144–160) [61]. Further, Prakash et al. investigated the ability of an ultrasensitive HBsAg assay to overcome the challenge of weak-reactives [49]. Of 248 samples testing positive with a standard assay, 180 (72.58%) were repeat reactive. Notably, 159 (64.11%) samples tested negative with the ultrasensitive assay (*p* < 0.0001). The standard and ultrasensitive assays had a concordance rate of 57.67%. With a much lower LoD compared to the standard assay (0.0046 IU/mL versus 0.021 IU/mL), the authors concluded that the ultrasensitive assay was better positioned to resolve weak reactive samples [49]. Further, between 1% and 48% of samples testing negative with standard HBsAg assays (lower LoD, 0.05 IU/mL) yield a positive result with ultrasensitive HBsAg assays (lower LoD, 0.005 IU/mL) [21].

False positive results are a common concern with HBsAg assays. A recent study of a standard HBsAg assay from Japan reported high false-positive rates of 33.1% in the HBsAg range of 0.005–0.049 IU/mL and 1.2% when HBsAg > 0.05 IU/mL [62]. Consequently, HBsAg weak-reactives/false positives require confirmatory testing, including neutralization tests, which incur extra time and resources [49,62].

Ultrasensitive HBsAg assays that have high specificity offer a potential solution by enhancing diagnostic accuracy and reducing the need for additional testing. However, greater sensitivity of an HBsAg assay is often achieved at the cost of specificity [48]. This could increase the likelihood of false-positive results under certain conditions. For instance, transient HBsAg positivity may occur due to passive transfer of the antigen following vaccination with a recombinant HBV vaccine, although this finding usually does not persist beyond 2 weeks [59]. False positivity may also occur in rare cases of tumors, such as parathyroid adenoma [63]. Cross-reactivity may arise in the presence of heterophilic antibodies, such as in cases of infection, transfusion, and systemic disease [63]. Moreover, isolated HBsAg seropositivity may occur in early infection, cases of HBV mutation in the S-region, autoimmune conditions, and reactivation of herpes virus or *Helicobacter pylori* infection [63]. Finally, confirming HBsAg loss may be difficult in the presence of low-level HBsAg expression driven by integrated HBV DNA [64]. Laboratory personnel and clinicians should remain vigilant regarding these factors that can significantly impact diagnostic interpretation and decision-making and cause social stigma to the patient. In such cases, confirmatory HBsAg testing can be performed to ensure accurate diagnosis.

Notably, Sickinger et al. [48] and Lou et al. [61] observed that the enhanced sensitivity of the investigated ultrasensitive HBsAg assay did not adversely impact its specificity. They observed 100% specificity in their studies, which may be attributed to the targeted refinements to design and process in ultrasensitive assays that help to simultaneously enhance both sensitivity and specificity [7,59]. This resulting balance can aid clinicians in making early and accurate decisions, reducing the need for unnecessary additional testing and treatment.

Table 4 and Table 5 present a comparative analysis of the sensitivity and specificity of ultrasensitive and standard HBsAg assays [3,10,48,61,65,66].

### 5.2. Clinical Utility Across the HBV Infection Spectrum, Including OBI and HBV-R

At present, clinical gaps persist in the detection of early and late acute HBV infection, as well as OBI [20]. With an exceptionally low LoD, ultrasensitive HBsAg assays hold vast potential for diagnosing asymptomatic HBV-infected patients in the low replication phase [3]. Coupled with sensitive HBV DNA assays, they could identify carriers of HBV and those at risk of liver-related complications and HBV-R as well as minimize risk of HBV transmission [60].

The period before HBsAg becomes detectable is the early window period, which can be shortened through enhanced HBsAg assay sensitivity [20]. Ultrasensitive HBsAg assays have been found to reduce the early window of detection by an average of 6.3 days compared to standard HBsAg assays [61]. Notably, Wong et al. observed that an ultrasensitive assay can detect HBsAg an average of 14 days earlier than standard assays [60].

Kuhns et al. assessed incremental detection by an ultrasensitive assay in 347 samples of acute infection and OBI from patients from the USA, South Africa, Spain, Cameroon, and Vietnam [65]. The assay showed improved detection of both early acute infection (nucleic acid testing [NAT] yield) and OBI samples, with incremental detection of 33.6% and 22.3%, respectively. Moreover, the assay improved the detection of HBsAg in the presence of anti-HBs. Eighteen of 86 (20.9%) NAT yield and OBI samples incrementally detected by the assay were anti-HBs-positive. Anti-HBs ≥ 0.01 IU/mL was present in 28.6% of samples testing positive with the assay and in 45.2% OBI samples testing negative with the assay. The authors speculated that anti-HBs > 0.3 IU/mL coupled with an extremely low viral load may result in limited HBsAg detection [65]. However, other research indicates that ultrasensitive assays can identify low HBsAg levels even when complexed with anti-HBs and in the presence of 2.5 IU/mL anti-HBs in CHB and OBI cases [67,68]. In a separate study, Kuhns et al. tested an HBsAg-anti-HBs panel using an ultrasensitive HBsAg assay and found that it could detect even low HBsAg levels in the presence of anti-HBs concentrations 250–400 times higher than those tolerated by current HBsAg assays [66]. However, the mechanisms underlying this performance of the tested ultrasensitive HBsAg assay remain sparsely discussed in literature.

Gupta et al. evaluated the performance characteristics of an ultrasensitive assay in 439 clinical samples from patients and healthy donors [3]. Notably, the assay demonstrated a lower LoD of 0.0033 IU/mL, with incremental detection of HBsAg in 11 additional samples (including 5 samples with OBI) compared to a standard HBsAg assay [3]. In another study by Steve et al., an ultrasensitive assay demonstrated an LoD of 0.0043 IU/mL [10].

Kuhns et al. further investigated the ability of an ultrasensitive assay to detect early and late acute infection and OBI [20]. Compared to a standard assay, the ultrasensitive assay increased detection of NAT yield samples (28/77, 36.4%), late acute infection (up to ≥13 days longer detection), and OBI (11/101, 10.9%). Genotypes A1, A2, B2, B4, C1, C2, C5, D3, E, and H were all adequately detected [20]. Similarly, Bourdin et al. observed the ability of an ultrasensitive assay in detecting weak positive samples, with improved sensitivity and specificity across varied genotypes [67]. They included 253 samples split into four panels: (1) routine prospectively screened serum samples (*n* = 196), (2) retrospective serum samples before HBV-R (*n* = 18), (3) OBI (*n* = 10), and (4) a selection of wild-type HBV genotype samples (*n* = 29) [67]. Although Panel 1 showed robust agreement between the ultrasensitive and standard assays, seven false positive samples with the standard assay tested negative with the ultrasensitive assay. This result was further corroborated with confirmatory testing. The ultrasensitive assay also identified one case of OBI. Interestingly, in Panel 2, four of 18 samples (22%) tested positive for HBsAg with the ultrasensitive assay. These findings indicate potential time savings of 1 to 6 months in diagnosing HBV-R, which could have significant clinical implications. In Panel 3, the 10 samples were previously shown to be HBsAg-negative and HBV DNA-positive. All samples were anti-HBc-positive and anti-HBs negative. The findings highlighted the ability of the ultrasensitive assay in detecting OBI. In Panel 4, the ultrasensitive assay detected all different genotypes with a greater tendency for detection compared to a standard assay; however, this difference was not significant. The authors concluded that the ultrasensitive assay was effective in resolving a high proportion of weak-reactive samples in HBV-R and OBI [67]. Consistent with these findings, Wong et al. observed that seroconversion sensitivity increased by 22–25% on using an ultrasensitive assay [60].

These findings highlight the significant clinical utility of ultrasensitive HBsAg assays in enabling early detection, identifying OBI, and improving monitoring of HBV-R, making them a potentially valuable tool in clinical practice.

### 5.3. Detecting Vaccine Breakthrough Infections

Although HBV vaccines are highly effective, breakthrough infections can still occur. In these cases, index samples often test negative for anti-HBc but positive for HBV DNA, even when protective levels of anti-HBs are present. HBsAg detection may be delayed or absent, and some breakthrough infections have been linked to HBsAg mutations [66].

Kuhns et al. found that an ultrasensitive HBsAg assay was more sensitive in detecting breakthrough infections than standard HBsAg assays or preS2 antigen and HBcoreAg testing [66]. Their study included serial samples from two commercially available plasma donor panels. The ultrasensitive assay detected panel 6272 at day 51, 43–46 days earlier than the comparator HBsAg assays [66].

### 5.4. Assessing Treatment Response and Functional Cure

The importance of attaining functional cure in chronic hepatitis B has continued to increase in recent years, with research showing the prognostic utility of HBsAg clearance. Several studies indicate that the HBsAg level at the time of stopping NA treatment is a strong predictor of whether HBV infection will remain inactive or relapse [69,70,71]. In one study, of 116 patients with HBsAg loss after treatment with entecavir, adefovir, lamivudine, and/or interferons, 18 (15.5%) tested positive with the ultrasensitive assay. Similarly, of 54 patients with spontaneous clearance, 15 (27.7%) tested positive with the ultrasensitive assay [10]. This assay demonstrated superior signal intensity compared to a standard assay across different phases of HBV infection, including samples with prozone effect. Further, it allowed for cost-effective in-house neutralization to confirm low HBsAg levels [10].

The global RETRACT-B study analyzed off-therapy outcomes after NA cessation to identify factors that could assist selection of patients for NA withdrawal [69]. At 4 years post-EoT, the cumulative probability of HBsAg loss at EoT was higher among patients >50 years than those <50 years; among Whites than Asians; and among those with HBsAg levels <100 IU/mL than between 100 and 1000 or >1000 IU/mL at EoT. Interestingly, 83.4% of the cohort had virologic relapse, 54.6% had clinical relapse, and 54.7% had started retreatment [69]. Additionally, a post hoc analysis of UMIN000001299 evaluated the efficacy of an ultrasensitive assay (lower LoD, 0.005 IU/mL) for samples of lymphoma patients whose HBV infection resolved with anti-CD20 antibody, rituximab-containing chemotherapy [72]. A positive result with the assay at baseline was an independent risk factor for HBV-R. The sensitivity of the standard (lower LoD, 0.05 IU/mL) and ultrasensitive assays at HBV-R were 18.2% and 77.3%, respectively, indicating the superior performance of the ultrasensitive assay [72].

Thus, ultrasensitive assays have an important role in predicting the possibility of functional cure during follow-up. This assertion is further strengthened by the findings of the phase 2 REP 401 study (NCT02565719), where serum HBsAg levels declined to <0.05 IU/mL during therapy (in 24 of 40 participants) and were maintained at <0.005 IU/mL until EoT [73]. Further, HBsAg levels declined below 0.005 IU/mL during follow-up in 16 of 40 participants [73]. These findings underline the effect of HBsAg clearance during and after nucleic acid polymer-based combination therapy and the role of HBsAg assays to quantify the efficacy of combination therapies targeting complete suppression of HBsAg and thereby, functional cure [73].

Further, these findings indicate that ultrasensitive assays are a huge leap forward for novel drug trials. They can help generate reliable data to determine the optimum time for stopping treatment and beginning retreatment, increasing confidence among clinicians and patients.

### 5.5. Performance Relative to NAT

Laboratory-based quantitative NAT assays have long been considered the standard-of-care assays for diagnosing and monitoring HBV DNA. However, few studies have directly compared the performance of ultrasensitive HBsAg assays with that of NAT. The available evidence suggests that ultrasensitive HBsAg assays approach the sensitivity of mini-pool NAT, with superior mutant and genotype detection [61]. Further, in seroconversion panels, while the number of days to the first confirmed result was less with an ultrasensitive assay than with a conventional assay, it was higher than that with NAT [60]. Reflecting the high diagnostic performance of these assays, the latest EASL guidelines (2025) also recommend using ultrasensitive HBsAg assays when NAT is unavailable [8].

## 6. Broader Implications of Ultrasensitive HBsAg Assays

### 6.1. Significance for the Immunosuppressed Population

Immunosuppressed individuals, including those undergoing treatment for malignancies, autoimmune disorders, chronic rheumatic diseases, or post-transplantation care, face a heightened risk of HBV-R, particularly if they have undetected chronic, resolved, or occult infection [74]. Mortality from HBV-R in patients receiving chemotherapy for hematological disorders has been found to range between 5 and 40%, whereas the incidence of HBV-R in HBsAg-positive individuals with systemic inflammatory diseases can reach up to 67.5% [75]. Recent research revealed that in cancer patients with persistent HBV infection treated with PD-1/PD-L1 inhibitors, the overall prevalence of HBV-R was 5% [76].

Despite the availability of vaccines, screening tests, and antiviral therapies, breakthrough infections and HBV-R remain serious complications, often leading to severe hepatitis, liver failure, and even death [74,77]. These findings highlight the increasing susceptibility of immunosuppressed persons, with the worldwide increase in HBV-R probably connected to the rising use of new immunosuppressive medications and inconsistent screening and management approaches [74].

In this setting, using highly sensitive diagnostic assays, such as ultrasensitive HBsAg assays, becomes crucial. Notably, an ultrasensitive HBsAg assay detected HBsAg 4 weeks before an HBV-R diagnosis in an individual receiving rituximab therapy [60]. Therefore, integrating an ultrasensitive HBsAg assay into routine screening programs may enhance prediction of HBV-R in immunocompromised individuals, including those requiring transplants or immunosuppressants, and decrease related complications and mortality.

### 6.2. Economic Impact

While NAT offers good specificity and stability, it requires specialized equipment and trained personnel. It can be technically complex, time consuming, and expensive, limiting its accessibility, especially in resource-constrained settings [8]. Ultrasensitive HBsAg assays can be particularly valuable where NAT is unavailable or infeasible, bridging the diagnostic gap between complex molecular platforms and less sensitive rapid tests in resource-limited environments [8].

Although laboratory-based, these assays show good alignment with select WHO REASSURED principles by offering high reliability, sensitivity, and analytical robustness. The use of ultrasensitive HBsAg assays across diverse clinical subgroups—stratified by age, race/ethnicity, baseline HBsAg level, genotype, and HBV S variants—has reduced misdiagnosis and the need for repeat testing [77,78]. These assays are positioned to effectively resolve weak reactive samples while demonstrating a good correlation with confirmatory/reflex tests and clinical disease [45]. This superior internal benchmarking can reduce the quantum of additional confirmatory testing, thereby also reducing the incurred costs [49]. By enhancing diagnostic efficiency and reducing unnecessary testing, ultrasensitive assays can also save valuable time, offering a cost-effective solution for laboratories and hospitals [67]. Such improvements could further increase the overall outcomes in addition to those already demonstrated with universal hepatitis B screening, which is estimated to save $262,857 (2020 USD) and help gain 135 quality-adjusted life years per 100,000 adults [79].

### 6.3. Role in Public Health

With increasing clinical trial data being generated on the enhanced performance of ultrasensitive HBsAg assays, their direct role in refining patient-centric treatment outcomes for HBV management is evident. Moreover, these assays can influence public health at various levels, from improving antenatal screening in pregnant mothers to HCC surveillance monitoring during and after cessation of treatment [80]. They can also be used to screen other high-risk populations, including immunocompromised and incarcerated individuals [18]. Moreover, the risk of transfusion-transmitted HBV infections is high if these patients serve as blood donors [3]. High-sensitivity HBsAg assays address this concern and enable blood screening safety [3]. Recent guidance from the U.S. Food & Drug Administration (FDA) states that HBsAg testing of donated blood is not necessary when it is tested for HBV DNA by NAT and for anti-HBc using FDA-approved screening tests. However, HBsAg testing of plasma donations should be continued as they are not tested for anti-HBc [81,82]. In many developing countries, NAT for HBV in donated blood is not mandatory, highlighting the need for ultrasensitive HBsAg assays to reduce risk of HBV transmission [67].

Holzmayer et al. showed that whole blood dried blood spot (DBS) is a feasible sample input for a specific HBsAg ultrasensitive assay, with no need for assay modification [83]. Therefore, detection of DBS samples with high-throughput serologic assay platforms may support global population surveillance programs [83].

## 7. Future Scope in HBsAg Testing

The widespread use of HBsAg as a biomarker for screening and assessing active disease is hindered by the limited availability of rapid diagnostic tests, particularly in resource-limited settings [84]. Rapid tests generally have lower sensitivity compared to high-sensitivity HBsAg assays, making them less suitable for monitoring treatment response [84]. DBS-based screening could enable rapid, accurate detection in large surveys where conventional draws are impractical or in resource-limited regions [85].

Further technological advances in HBsAg assays should focus on universal screening and enhancing detection in asymptomatic cases of HBV. Strengthening public health initiatives could promote wider adoption of ultrasensitive assays and enhance case identification and linkage to care, accelerating progress toward HBV elimination, especially in endemic and resource-limited regions.

Although quantitative HBsAg testing allows assessment of cccDNA and/or integrated DNA transcriptional activity, there remain challenges in distinguishing between the two sources of surface antigen [86,87]. Further research on distinguishing HBsAg production originating from cccDNA versus integrated DNA could offer insight into the durability of a functional cure and help guide treatment adjustments for patients with CHB [86,87].

## 8. Conclusions

Ultrasensitive HBsAg assays demonstrate a substantial development in conquering screening, diagnostic, and monitoring constraints as well as enabling safe blood donation. Research indicates the enhanced performance of ultrasensitive assays in early HBV detection, including patients testing negative with conventional HBsAg assays. By identifying minimal antigen levels, these assays enhance diagnostic accuracy, particularly for patients with occult infection. As a reliable diagnostic tool, ultrasensitive assays enable clinicians to plan individualized treatment timelines by providing (i) granular data on antigen decline over time, (ii) reliable prognosis for estimating the probability of achieving sustained remission, and (iii) evidence for extending or discontinuing therapy, especially in patients with borderline low HBsAg levels. These assays also play an important role in treatment monitoring, supporting clinical decisions regarding long-term therapy discontinuation.

In conclusion, with their increased sensitivity and predictive value, these assays greatly assist in improving HBV management, directing treatment cessation plans, and decreasing the risk of recurrence.

## Figures and Tables

**Table 1:** Interpretation of key serological markers of HBV infection.

HBsAg	Total Anti-HBc	IgM Anti-HBc	Anti-HBs	Interpretation
Negative	Negative		Negative	Susceptible, never infected (if no record of vaccine series completion) Anti-HBs concentrations may decline among vaccine responders. Individuals with record of vaccine series completion typically do not require revaccination, except specific groups like patients on hemodialysis or healthcare personnel [18,19]
Positive	Positive	Positive	Negative	Acute infection IgM anti-HBc may also be positive in chronic infection during severe infection flares or reactivation [18,19]
Positive	Positive	Negative	Negative	Chronic infection [18,19]
Negative	Positive	Negative	Positive	Resolved infection, immunity achieved [18,19]
Negative	Positive		Negative	Possible interpretations: Resolved infection, anti-HBs levels have waned OBI Passive transfer of anti-HBc to infant born to HBsAg-positive mother False positive, patient susceptible Mutant HBsAg strain not detected by laboratory assay [18,19]
Negative	Positive	Positive	Positive	Recent infection, recovered, immunity achieved [18,19]
Negative	Negative	Negative	Positive	Immunity due to vaccination (if record of vaccine series completion) Immune if anti-HBs concentration > 0.01 IU/mL after vaccine series completion [18,19]

anti-HBc, antibody to HBV core antigen; anti-HBs, antibody to HBV surface antigen; HBsAg, HBV surface antigen; HBV, hepatitis B virus; IgM, immunoglobulin M; OBI, occult HBV infection.

**Table 2:** Types of HBV cure.

Type	Description
Sterilizing/complete cure [35,36]	Undetectable serum HBsAg and elimination of HBV DNA, including intrahepatic cccDNA and integrated genomic HBV DNA
Functional cure [35,36,37]	Sustained HBsAg loss and undetectable HBV DNA in serum (6 months post-treatment), with or without seroconversion to anti-HBs, resolution of the residual liver injury, and persistence of low levels of intrahepatic cccDNA and HBV DNA integration
Partial cure [35,36]	Less stringent endpoint, refers to detectable HBsAg but persistently undetectable serum HBV DNA after completing a finite course of treatment

cccDNA, covalently closed circular DNA; HBsAg, HBV surface antigen; HBV, hepatitis B virus.

**Table 3:** Comparative analysis of HBsAg assay platforms.

Platform	Sensitivity	Specificity	Throughput	Operational Feasibility
Lateral flow assay [52,53]	Moderate-high	Moderate	Single-sample assays with results in ~15–30 min	Portable one-step tests No specialized equipment needed Suited to point-of-care testing Limited sensitivity may fail to identify early infection Visual readout carries risk of misinterpretation
ELISA [52,54,55,56,57,58]	Moderate-high	High	Batch testing on 96-well plates; hundreds of samples per run	Requires specialized lab equipment Techniques are relatively simple and low-cost
CLIA [3,10,57,58]	Very high	Very high	Fully automated analyzers can process hundreds of tests per hour	Fully automated Requires specialized lab equipment Closed systems Minimal hands-on time

CLIA, chemiluminescent immunoassay; ELISA, enzyme-linked immunoassay.

**Table 4:** Sensitivity of ultrasensitive HBsAg assays.

Study	Comparison of Ultrasensitive vs. Standard HBsAg Assays
Lou et al. [61]	Analytical sensitivity: 0.005 IU/mL; 3.86- to 14.54-fold more sensitive (standard assays, 0.02–0.07 IU/mL) Reduced early window period by 6.3 days Better detection of mutant specimens 2.4- to 9.3-fold higher sensitivity for genotype detection Seroconversion panels: 191 vs. 144–160 (standard assays) of 364 panels detected
Sickinger et al. [48]	Clinical sensitivity: 100% vs. 99.78% (standard assay) Reduced time to first repeat reactive and confirmed result in 75% samples Better detection of mutant specimens
Kuhns et al. [66]	Detection of serological panels 43–46 days earlier Greater sensitivity in detecting HBsAg in the presence of anti-HBs
Kuhns et al. [65]	Increased detection of NAT yield (131 samples) by 33.6% (44/131) Increased detection of OBI (188 samples) by 22.3% (42/188)
Gupta et al. [3]	100% concordance between ultrasensitive and standard HBsAg assays among different genotypes Incremental detection of HBsAg: 11 additional samples (including 5 samples with OBI)
Steve et al. [10]	Increased detection of HBsAg in treated patients with apparent HBsAg loss (116 patients) by 15.5% (18/116) Increased detection of HBsAg in patients with apparent HBsAg loss with spontaneous clearance (54 patients) by 27.7% (15/54)

anti-HBc, HBV core antigen; anti-HBs, hepatitis B surface antibody; CI, confidence interval; HBsAg, HBV surface antigen; HBV, hepatitis B virus; LoD; limit of detection; NAT, nucleic acid testing; OBI, occult HBV infection.

**Table 5:** Specificity of ultrasensitive HBsAg assays.

Study	Parameter	Specificity of Ultrasensitive HBsAg Assay	Remarks
Lou et al. [61]	Clinical specificity (10,633 blood donor specimens)	100%	Standard HBsAg assays: 99.95–99.99% Specificity retained despite improved sensitivity
	Clinical specificity (8439 diagnostic samples)	99.98%	Standard HBsAg assays: 99.86–99.93%
Sickinger et al. [48]	Clinical specificity (6618 blood donor specimens)	100%	Standard HBsAg assays: 99.2–99.6% Increased sensitivity of the investigational assay was not found to impact specificity.
	Clinical specificity (450 known positive samples)	100%	Standard HBsAg assays: 100%
Gupta et al. [3]	100 confirmed positive samples	100%	Standard HBsAg assay: 100%

HBsAg, HBV surface antigen.

## Data Availability

The original contributions presented in this study are included in the article. Further inquiries can be directed to the corresponding author.

## Abbreviations

The following abbreviations are used in this manuscript:

AASLD	American Association for the Study of Liver Diseases
Anti-HBc	HBV core antigen
Anti-HBs	Hepatitis B surface antibody
cccDNA	Covalently closed circular DNA
CHB	Chronic hepatitis B
CI	Confidence interval
CLIA	Chemiluminescent immunoassay
DBS	Dried blood spot
DNA	Deoxyribose nucleic acid
EASL	European Association for the Study of the Liver
ELISA	Enzyme-linked immunoassay
EoT	End of treatment
FDA	U.S. Food & Drug Administration
HBeAg	HBV e antigen
HBsAg	HBV surface antigen
HBV	Hepatitis B virus
HBV-R	HBV reactivation
HCC	Hepatocellular carcinoma
IgM	Immunoglobulin M
LoD	Limit of detection
NA	Nucleos(t)ide analog
NAT	Nucleic acid testing
OBI	Occult HBV infection
PCR	Polymerase chain reaction
PD-1	Programmed death protein 1
PD-L1	Programmed death ligand-1
WHO	World Health Organization
